# Identification of risk factors in minimally invasive surgery: a prospective multicenter study

**DOI:** 10.1007/s00464-016-5248-4

**Published:** 2016-10-31

**Authors:** Sara R. C. Driessen, Evelien M. Sandberg, Sharon P. Rodrigues, Erik W. van Zwet, Frank Willem Jansen

**Affiliations:** 1 Department of Gynaecology, Section Minimally Invasive Surgery, Leiden University Medical Center, PO Box 9600, 2300 RC Leiden, The Netherlands; 2 Department of Medical Statistics, Leiden University Medical Center, PO Box 9600, 2300 RC Leiden, The Netherlands; 3 Department BioMechanical Engineering, Delft University of Technology, PO Box 5, 2600 AA Delft, The Netherlands

**Keywords:** Patient safety, Laparoscopy, Risk factor, Safety, Hysterectomy

## Abstract

**Background:**

Since the introduction of minimally invasive surgery (MIS), concerns for patient safety are more often brought to the attention. Knowledge about and awareness of patient safety risk factors are crucial in order to improve and enhance the surgical team, the environment, and finally surgical performance. The aim of this study was to identify and quantify patient safety risk factors in laparoscopic hysterectomy and to determine their influence on surgical outcomes.

**Methods:**

A prospective multicenter study was conducted from April 2014 to January 2016, participating gynecologists registered their performed laparoscopic hysterectomies (LHs). If deemed necessary, gynecologists could fill out a checklist with validated patient safety risk factors. Association between procedures with and without an occurred risk factor(s) and the surgical outcomes (blood loss, operative time, and complications) were assessed, using multivariate logistic regression and generalized estimation equations.

**Results:**

Eighty-five gynecologists participated in the study, registering a total of 2237 LHs. For 627(28 %) procedures, the checklist was entered (in total 920 items). The most reported risk factors were related to the surgeon (19.6 %), the surgical team (14.4 %), technology (16.6 %), and the patient (26.8 %). The procedures where a risk factor was registered had significantly less favorable outcomes, higher complication rate (10.5 vs. 4.8 % (*p* = 0.002), longer operative time [114 vs. 95 min (*p* < 0.001)], and more blood loss [110 vs. 168 mL (*p* = 0.047)], which was mainly due to the technological and patient-related risk factors.

**Conclusion:**

Technological incidents are the most important and clinically relevant risk factors affecting surgical outcomes of LH. Future improvements of MIS need to focus on this. As awareness of safety risk factors in MIS is important, embedding of a safety risk factor checklist in registration systems will help surgeons to evaluate and improve their individual performance. This will inherently improve the surgical outcomes and thus patient safety.

Since the introduction of minimally invasive surgery (MIS) in daily surgical practice, patient safety issues have increasingly received attention. Implementation of new technologies in surgery is a challenge for practicing surgeons, especially when it comes to complex procedures such as MIS. In general, MIS requires a more demanding work environment compared to conventional surgery, and in order to facilitate the surgeon in this, a fast development of new medical devices is observed [1]. In contrast to the introduction of newly developed drugs, new devices are mostly introduced into the operating room without proper evidence regarding their benefit and safety. This can potentially lead to patient safety issues in daily clinical practice, as also seen after the wide introduction of the laparoscopic power morcellator; years after this introduction, the US Food and Drug Administration (FDA) issued a statement discouraging the use of power morcellation in the majority of women undergoing hysterectomy or myomectomy for uterine fibroids due to the potential risk of upstaging of uterine sarcoma [2].

Besides improper introduction of new technologies, limited experience and skills of the surgeon are considered to be important risk factors in MIS [3]. In addition, also communication and environmental failures occur commonly during surgical procedures and are recognized as risk factors regarding patient safety [1, 4]. Knowledge about and awareness of these patient safety risk factors are crucial to improve and enhance the surgical team, the environment, and finally surgical performance. However, it is not known whether and how these validated risk factors directly affect surgical outcome. In order to improve the surgical process, insight into the occurrence of events as potential risk factors and their consequences are required. Laparoscopic hysterectomy (LH) is the most performed advanced laparoscopic procedure in gynecological surgery [5]; therefore, this procedure is ideal for further analyses. The aim of this multicenter prospective study was to identify and quantify patient safety risk factors in LH and to assess their influence on surgical outcomes.

## Materials and methods

During this prospective multicenter study, all gynecologists performing advanced MIS (regarding the ESGE classification [6]) were asked to register their consecutive LHs from April 2014 to January 2016 in a secured web-based application.

During initial registration, gynecologists were asked to enter the number of LHs performed yearly (their annual surgical volume), the total amount of LH performed during their career (their experience), and the number of years they were performing LHs. After initial registration, the application was available 24/7 for the registration of all consecutive performed LHs.

After entering the procedure data, the gynecologist could optionally enter a checklist with validated patient safety risk factors and observations, which could have potentially influenced the outcome of the procedure (Table 1). The risk factor checklist was developed based upon previous research [3]. A brief description of every domain and risk factor was easily available by the use of information pop-ups. A free text option was available to write additional comments.

**Table 1:** Used patient safety risk factor checklist with number and percentage of entered items per domain

Domain (detailed description)	Number of entered domains (%, and % of total procedures *N* = 2237)	Detailed risk factors per domain	Number of entered detailed options
Surgeon (functioning of the surgeon)	164 (19.6; 7.3)	Lack of experience (of surgeon or resident)	141
		Lack of technical skills (of surgeon or resident)	27
		Lack of leadership	2
Surgical team (functioning of the scrub or circulating nurse)	120 (14.4; 5.4)	No qualified staffing (e.g., student/pupil because of shortage of staff or unqualified staffing)	25
		Lack of experience of the scrub nurse (concerning this procedure)	78
		Lack of knowledge of the procedure of scrub nurse	26
		Lack of experience of circulating nurse	37
Technology (availability and functioning of equipment and instruments)	139 (16.6; 6.2)	Instrument(s) not present or available	18
		Instrument(s) do(es)n’t work properly	75
		It is not known how to handle instruments (either surgeon or scrub nurse)	5
		Equipment is not present	4
		Equipment does not work properly	19
		Limited vision (e.g., because of condensation and/or smoke)	31
		It is not known how to handle equipment (either surgeon or scrub nurse)	5
Social interaction (teamwork and communication)	9 (1.1; 0.4)	Poor communication between OR team members (e.g., misunderstandings)	5
		Failure of professional communication (either verbal or nonverbal)	1
		Poor collaboration between OR team members	3
Environment (potentially cause distraction or disruptions of the surgical process)	21 (2.5; 0.9)	Distractions (e.g., telephone calls, case irrelevant conversations, door movements)	10
		Disruption of the surgical process (surgical process has to be interrupted because of distractions)	7
		Too many people in the OR	4
Patient (patient-related risk factors)	224 (26.8; 10)	Severe adhesions	182
		Unexpected comorbidity, please specify (e.g., unknown bleeding disorder (e.g., v Willebrand disease, hemophilia))	57
Fallibility (factors that influence the fallibility of the surgeon)	11 (1.3; 0.5)	Moment of day surgery takes place (e.g., during evening or night shifts)	1
		Perceived high workload	3
		Fatigue of the surgeon	7
Safety (compliance or safety protocols)	1 (0.1; 0.04)	Poor compliance of briefing procedure	0
		Poor compliance of debriefing procedure	1
		Poor compliance of (surpass) checklist (if applicable)	0
Anesthesiology	30 (3.6; 1.3)	Anesthesiology-related problems	30
Other	116 (13.9; 5.1)	Free text option, please specify	116
Total	835		920

The following patient characteristics were registered: age, BMI (kg/m^2^), uterine weight, number of previous abdominal surgeries defined as laparotomy (including cesarean section) or therapeutic laparoscopy, and the presence and stage of endometrioses (stage 1 minimal, stage 2 mild, stage 3 moderate, and stage 4 severe, as defined by the American Society for Reproductive Medicine [7]). Additionally, the surgical outcomes collected included the type of hysterectomy (total laparoscopic hysterectomy (TLH), supracervical laparoscopic hysterectomy (SLH), laparoscopic-assisted vaginal hysterectomy (LAVH), and robotic hysterectomy), intra-operative blood loss (milliliters, collected in containers and directly measured after surgery), operative time, and complications. Operative time was defined as the number of minutes between first incision and the final stitch. Complications were registered according to the classification of the Dutch Society of Obstetrics and Gynecology [8], including infection (local, organ, and systemic), injury (vascular, bowel, bladder, and ureter), wound dehiscence, hemorrhage (>1000 mL, postoperative bleeding), thromboembolism, dysfunction (urinary retention, incontinence, ileus, liver, kidney), systemic (medication error, adverse drug reaction), technical (failed procedure, corpus alienum), reactive conversions (as defined by Blikkendaal et al. [9]), and other (not specified). The postsurgical follow-up period lasted for 6 weeks after discharge. After the 6 weeks, gynecologists received an automatic reminder from the application to register any possible postoperative complication. All surgical outcome data were mandatory items to register in the web-based application.

Since only limited anonymous patient data were requested, our Institutional Review Board at Leiden University Medical Center exempted this study (C14.002) from approval.

### Data analysis

For the statistical analysis, SPSS version 20.0 (SPSS Inc, Chicago, IL) was used. The number of entered risk factor was summed per domain and per detailed risk factor (Table 1). Mean values were calculated and shown with their standard deviation (SD). Patient characteristics and surgical outcomes were compared between two groups: LHs with entered risk factor(s) and LHs without entered risk factor(s) (Table 2). Multivariate logistic regression was used for risk adjustment in assessing associations between procedures with and without an entered risk factor checklist and surgical outcomes. Variables used in this model included BMI, previous abdominal operations, the presence of endometriosis, type of LH, uterine weight, operative time, blood loss, and complications (Table 2). A sub-analysis was performed comparing entered risk factor per domain and surgical outcomes (Table 3). The influence of surgeon’s volume, experience, and years of experience on the entering of a risk factor checklist were calculated using binary logistic regression analysis.

**Table 2:** Patient characteristics and surgical outcomes for LHs with entered risk factor and without entered risk factor

Variable	Total *N* = 2237	LHs with entered risk factor *N* = 627	LHs without entered risk factor *N* = 1610	*p* value	Odds ratio (95 % CI)
Age ± SD	48.8 ± 11.6	48.6 ± 11.4	48.9 ± 11.6	0.749	1.002 (0.991–1.012)
BMI kg/m^2^ ± SD	28.3 ± 6.1	29.0 ± 6.3	28.0 ± 6.02	0.249	1.010 (0.993–1.026)
Previous abdominal surgery				<0.001	–
None %	56.0	49.4	58.5		
1 %	25.8	26.7	25.5		
2 %	11.3	13.3	10.5		
>2 %	6.9	10.5	5.5		
Type LH				0.449	–
TLH %	91.2	91.1	91.3		
SLH %	4.5	4.8	4.3		
LAVH %	3.2	3.0	3.3		
Robotic %	1.1	1.1	1.1		
Uterus weight gr ± SD	217.3 ± 206.3	240.5 ± 234.7	209.1 ± 193.2	0.079	0.999 (0.999–1.000)
Endometriosis %	15.3	19.9	13.5	0.562	0.915 (0.667–1.236)
Blood loss mL ± SD	126.2 ± 164.0	167.6 ± 201.6	110.1 ± 143.7	0.047	1.001 (1.000–1.002)
Operative time min ± SD	100.6 ± 39.0	114.3 ± 41.6	95.3 ± 36.6	<0.001	1.014 (1.009–1.019)
Complication %	6.4	10.5	4.8	0.002	0.548 (0.376–0.797)

*LH* laparoscopic hysterectomy, *TLH* total laparoscopic hysterectomy, *SLH* supracervical laparoscopic hysterectomy, *LAVH* laparoscopic-assisted vaginal hysterectomy, *RALH* robotic-assisted laparoscopic hysterectomy

**Table 3:** Difference in surgical outcomes of LHs with and without an entered risk factor checklist stratified per safety domain

Safety domain entered checklist?	Blood loss ml ± SD	*p* value	Operative time min ± SD	*p* value	Complications	*p* value
Surgeon						
Yes (*n* = 164)	135.0 ± 156.5	0.879	109.8 ± 26.7	0.408	8.5 %	0.445
No (*n* = 2073)	125.5 ± 164.6		99.8 ± 39.7		6.3 %	
Surgical team						
Yes (*n* = 120)	148.8 ± 203.3	<0.001	107.4 ± 37.8	<0.001	6.7 %	0.032
No (*n* = 2117)	124.9 ± 161.5		100.2 ± 39.0		6.4 %	
Technology						
Yes (*n* = 139)	202.6 ± 286.3	<0.001	126.9 ± 53.1	<0.001	12.2 %	<0.001
No (*n* = 2098)	121.1 ± 151.3		98.9 ± 37.2		6.1 %	
Social interaction						
Yes (*n* = 9)	141.7 ± 106.1	0.428	129.6 ± 30.8	<0.001	11.1 %	0.242
No (*n* = 2228)	126.1 ± 164.2		100.5 ± 39.0		6.4 %	
Environment						
Yes (*n* = 21)	200.5 ± 206.6	0.005	126.7 ± 48.1	<0.001	9.5 %	0.554
No (*n* = 2216)	125.5 ± 163.5		100.3 ± 38.8		6.4 %	
Patient						
Yes (*n* = 224)	213.5 ± 244.9	<0.001	120.6 ± 48.7	<0.001	14.3 %	<0.001
No (*n* = 2013)	116.5 ± 149.4		98.4 ± 37.1		5.6 %	
Fallibility						
Yes (*n* = 11)	101.8 ± 72.2	0.531	113.5 ± 38.5	0.034	9.1 %	0.358
No (*n* = 2226)	126.3 ± 164.4		100.5 ± 39.0		6.4 %	
Safety						
Yes (*n* = 1)	Na		Na		Na	
No (*n* = 2236)	Na		Na		Na	
Anesthesiology						
Yes (*n* = 30)	154.7 ± 149.4	0.293	114.5 ± 39.0	0.001	10.0 %	0.357
No (*n* = 2207)	125.8 ± 164.2		100.4 ± 39.0		6.4 %	

*Na* not applicable

To account for the clustering of data from multiple entered procedures and risk factor checklists by a single surgeon, generalized estimation equations were used for all analyses. Ninety-five percent confidence intervals were calculated of all odd ratios. Statistical significance was defined as a *p* value <0.05.

## Results

During the study period, a total of 85 gynecologists participated and entered their performed LHs. Mean (SD) LH experience (total amount of performed LH during their career) of the surgeons was 177 (173), with a range of 800 procedures. A total number of 2237 LH procedures were entered, and for 627 (28 %) procedures, the risk factor checklist was filled in. Because more than one risk factor could be entered per procedure, a total of 920 patient safety risk factors were registered. All entered risk factor are depicted in Table 1, subdivided by domain. The most frequently reported risk factor domains were surgeon (19.6 % and in 7.3 % of all procedures), surgical team (14.4 % and in 5.4 % of all procedures), technology (16.6 % and in 6.2 % of all procedures), and patient-related risk factors (26.8 % and in 10 % of all procedures) (Table 1). Regarding the domain “surgeon,” lack of experience (of surgeon or resident) was mainly reported, i.e., 141 times (15.3 % of all entered items). Furthermore, lack of experience/knowledge of the scrub/circulating nurse was also considered one of the main potential risk factors, reported in total 141 times (15.3 % of all entered items). Registered technology-related events included mainly the improper functioning of instrument(s) and/or equipment and were reported in total 94 times (10.2 % of all entered items). Patient-related factors such as unexpected severe adhesions were mentioned 182 times (19.8 % of all entered items). Social interaction including teamwork and professional communication was entered 9 times (1 % of all entered items). Other patient safety risk factors with low count of events were environment (2.2 %), fallibility of the surgeon (0.5 %), and lack of compliance to the safety protocols (1.2 %). Anesthesiological-related issues were reported in 30 of the procedures (3.2 % of all entered items).

In 116 procedures, the free text option was filled out. The main issues reported were patient-related issues (e.g., morbid obesity, adhesions, previous operations, endometriosis, large uterus, and fibroids), together with logistical and setup problems (e.g., “had to wait for assistance,” “testing new equipment in new theater,” “procedure was part of a training course”).

Table 2 shows patient characteristics and surgical outcomes of entered procedures and the differences between procedures with (*n* = 627) and without (*n* = 1610) an entered risk factor checklist. There were no significant differences in patient characteristics between the two groups with the exception of previous abdominal surgery (*p* < 0.001), with a higher rate in the LH group where a risk factor checklist was entered. For all reported surgical outcomes, a significant difference was observed in favor of the procedures where no risk factors occurred: complications 10.5 versus 4.8 % (*p* = 0.002), blood loss 110.1 versus 167.6 mL (*p* = 0.047), and operative time 114.3 versus 95.3 min (*p* < 0.001).

Table 3 shows the difference in surgical outcomes stratified per entered risk factor domain.

When technological-related risk factors were registered, all surgical outcomes were significantly less favorable (*p* < 0.001 for blood loss, operative time, and complications). This also was found for the procedures with risk factors related to the surgical team (e.g., no qualified staffing, lack of experience/knowledge of the scrub/circulating nurse) and to patient-related issues (especially adhesions). It appeared that for procedures where surgeon-related risk factors occurred (e.g., lack of experience and/or lack of technical skills), no significant difference was observed in surgical outcomes compared to procedures where no risk factor occurred.

The experience of the surgeon was not correlated to the number of registered risk factor checklist of the surgeon, *p* = 0.425 (95 % CI = 0.998–1.001). A similar result was seen for surgeon’s volume and years of experience, respectively *p* = 0.936, (95 % CI 0.987–1.014) and *p* = 0.085 [95 % CI = 0.999–1.015)].

## Discussion

In this prospective cohort study, 85 gynecologists entered their LHs, and when deemed necessary, they could additionally fill in a risk factor checklist. In 28 % of LHs, surgeons entered at least one patient safety risk factor. We observed less favorable surgical outcomes in the group LHs where a risk factor checklist was registered (Table 2). Patient-related risk factors and technological-related problems were listed as most important risk factor during LH, affecting negatively all surgical outcomes (Table 3). The lack of proper functioning equipment and instruments in the surgical field is well known to be associated with an increased risk of incidents [10]. In our study, 6.2 % of all registered procedures encountered technological problems. This percentage is considerably lower compared to previous studies, as Wubben et al. [11] found equipment-related incidents in 16 % of observed surgeries and Verdaasdonk et al. [12] observed technical incidents in 87 % of recorded laparoscopic cholecystectomies. However, these percentages are not comparable with our study, as they focused on technological incidents counted by direct observations or video observations. In our study, the registered events were entered by the surgeon him/herself, which makes these events clinically more relevant, and the event had to be serious enough for the surgeon to remember and register it afterward, especially since it might influence their surgical outcomes. Therefore, our number could be an underestimation of the actual percentage of occurred risk factors.

We observed that the occurrence of patient-related risk factors, such as adhesions, is of significant influence on all surgical outcomes (Table 3). We consider patient-related risk factors of a different nature compared to the other registered risk factors, for example as doctors cannot influence comorbidity of a patient (e.g., extent of adhesions and obesity) [13]; however, we do have a responsibility for technological issues or surgical team-related problems, and these are therefore important targets for future improvements regarding patient safety.

It is notable that surgeons criticized their selves (i.e., “functioning of the surgeon”) in 20 % of the registered risk factors. Surprisingly though, our data showed that the occurrence of these surgeon-related risk factors did not affect any surgical outcomes (Table 3). Yet, the occurrence of risk factors relating to the surgical team (i.e., lack of experience/knowledge of scrub/circulating nurse) did significantly affect surgical outcomes. Although it can be questionable whether a difference of 20–30 mL blood loss truly is clinically relevant (Table 3), it could indicate that the surgical team in its entirety is more important to surgical outcomes than previously thought [3]. Therefore, it seems obvious to assume that a dedicated and experienced surgical team will lead to increased efficiency, better communication, and inherently enhance patient safety. Still, we need to emphasize that the primary responsibility for a procedure and its outcomes lies in the hands of the (primary) surgeon and not the other members of the surgical team.

It has been shown that when a laparoscopic procedure is performed under distracting conditions, performance could be directly affected [14]. Our results showed that the effect of environmental events seems to be a minor subject since this domain was only entered 21 times, corresponding with <1 % of all procedures (Table 1). However, the occurrence of environmental risk factors adversely affected the outcomes blood loss and operative time (Table 3). This suggests that when an environmental event is clinically relevant and significant enough to be noticed, it could negatively influence outcomes. This observation emphasizes the clinical impact of the environment as also shown in previous studies [1, 4].

Since the development of the time-out protocol by the World Health Organization (WHO) [15], multiple publications demonstrated that the use of this protocol improves patient outcomes, teamwork, and communication [16]. In our study, the domain of safety (e.g., poor compliance of safety protocols) is only mentioned once. Therefore, we can conclude that the implementation of this briefing is well established and (inter) nationally accepted.

A potential limitation of our study is that it is conceivable that surgeons will enter more risk factor items when they performed a procedure with unfortunate outcomes, in order to justify their suboptimal performances. This could potentially lead to reporting bias. To correct for these limitations, we used generalized estimation equation to account for the clustering of data by a single surgeon.

Technological problems are the most relevant and important patient safety risk factors, and future improvements need to focus on this to enhance quality and safety of MIS. It is not acceptable that nowadays technological problems are still such a major patient safety issue in these modern times, and a concise training and/or briefing for the entire surgical team should be mandatory when new devices are introduced. Evidence showed that most technological issues can be solved with decent preparation and more attention to technology during briefing [1, 10].Our risk factor checklist can be seen as an individual guidance tool, for instance when the performance of a surgeon is consistently suboptimal. The use of the current checklist allows individual reflection and will potentially help to improve individual performance [16], and this will inherently increase awareness and insight in risk factors in MIS.
